# How to Give Digitalis

**Published:** 1900-09-08

**Authors:** 


### HOW TO GIVE DIGITALIS.

In a clinical lecture on the treatment of diseases of the heart, recently delivered at Guy's Hospital, Dr. Hale White gave various hints as to how to give digitalis. One formula which he recommended was as follows:—

℞ Tinct. digitalis, fl. dr. 3.
Tinct. nucis vom., fl. dr. 1½.
Caffein, gr. 50.
Sodii salicylatis, gr. 25.
Syrupi aurantii floris, fl. oz. 1.
Aquam ad., fl. oz. 10.
Fiat mistura.

One ounce of this mixture was to be taken thrice a day. Some people might say that theoretically caffein ought not to go with digitalis, for caffein did not slow the heart or prolong the diastole, nor did it belong to the digitalis group of drugs. But it increased the cardiac force, and experience had shown clinically that it went very well with digitalis. It helped its diuretic action, and, in so doing, it did not irritate the kidneys as so many other diuretics did. Some, again, might think that if caffein were to be given it would be better to give the citrate; but citrate of caffein, although soluble, might be precipitated when the solution containing it was stirred. It remained, he said, in solution for a time, but when the bottle was shaken the precipitate fell, and the patient might use strong language about the doctor who wrote out such a prescription. It was safer to give caffein, which was easily soluble in water if salicylate of sodium were added to it in the above proportion. The nux vomica acted as a cardiac stimulant too. It was to be noted that, although the above was a full dose of digitalis, it was often necessary to use more than the amount given in this prescription. As a rule, in fact, digitalis was not given sufficiently freely, and usually the mixture above prescribed would require to be given oftener than three times a day. Dr. Hale White warned his hearers against mixing citrate of caffein with salicylate of sodium, which, he said, formed a voluminous precipitate, the bottle becoming filled with a compound which it was almost impossible to pour out at all.

Another excellent way of giving digitalis was as the famous combination known as the Guy's pill. One pill contained a grain each of mercurial pill, powdered squill, and powdered digitalis leaves, and four grains of green extract of hyoscyamus. The only practical objection to this combination was that the digestive system of the patients might be such that they could not dissolve the pill. But still it was, without doubt, one of the best preparations of digitalis. The object of the squill was that it acted very like the digitalis, and also as a diuretic; while the reason of giving the mercury was that it was a well-known fact that its combination with the digitalis considerably increased the diuretic action of the latter. Unfortunately, digitalis often made the patient sick, and if that should be the case the next best drug to try was strophanthus. But this was not a complete substitute for digitalis, in that it did not contract the vessels all over the body, so that one did not get the same help from it in the circulation as one got in the case of digitalis. But strophanthus also might make the patient sick, in which case the next drug to try was the tincture of convallaria majalis, the lily of the valley, and if this failed then chloride of barium in half-grain doses might be given.

